# Paranoia and social anxiety: Structural and temporal relationships across adolescence

**DOI:** 10.1002/jcv2.70181

**Published:** 2026-10-12

**Authors:** Jessica L. Kingston, Björn Schlier, Richard P. Bentall, Christopher D. J. Taylor, Eleanor Leigh

**Affiliations:** ^1^ Department of Psychology Royal Holloway University of London Egham UK; ^2^ University of Wuppertal Wuppertal Germany; ^3^ School of Psychology, Faculty of Science University of Sheffield Sheffield UK; ^4^ Research and Innovation Pennine Care NHS Foundation Trust Ashton‐under‐Lyne UK; ^5^ Department of Experimental Psychology University of Oxford Oxford UK

**Keywords:** accelerated cohort design, adolescence, longitudinal, paranoia, social anxiety

## Abstract

**Background:**

Social anxiety (SA) and paranoia (PA) are commonly experienced during adolescence, but the nature of their co‐development is largely unknown. Greater clarity could significantly advance our understanding of both conditions, and of their progression into serious mental illness.

**Methods:**

Utilising the Neuroscience in Psychiatry Network 2k cohort data, an accelerated cohort design study in 14–22 year olds, pre‐registered hypotheses examined: (1) via measurement models, whether SA and PA were statistically distinguishable; (2) whether SA at a given time‐point was significantly associated with subsequent PA at the next time point; and (3) common co‐developmental trajectories through adolescence (e.g., low in both; high SA, low PA, etc.).

**Results:**

(1) Measurement models indicated that two and three factor models, with SA and PA as related but separate constructs, best fit the data across all age groups, (2) in cross‐lagged panel analysis, SA was a more significant predictor of subsequent (approximately 1 year later) PA than vice versa; (3) Six common patterns in how SA and PA developed together over time (i.e., co‐developmental trajectories) were detected, whereby low levels of each were most common, followed by clusters with elevated SA but not PA, followed by mid and descending SA and mid PA, or elevated levels on each.

**Conclusion:**

SA and PA are commonly comorbid in adolescence. More evidence was obtained for the *SA‐fuelling‐PA* hypothesis than vice versa, but nonetheless we also detected a *PA‐to‐SA* pathway in the youngest age group which requires further investigation. Understanding the social and cognitive factors that account for escalation from SA into PA is a key future direction.

## Key Points

**What's Known?**

- Paranoia (PA) and social anxiety (SA) commonly co‐occur in adolescents.

**What's New?**

- Over time, SA preceded and predicted future PA. However, the reverse was found in younger adolescents.
- Mulitple distinct co‐developmental pathways were identified, varying by degree of commonality.

**What's Relevant?**

- Improved early identification of trajectories, their mechanisms, and impacts, is vital.
- We need to assess for PA in adolescents, especially those with SA.

## INTRODUCTION

Socially oriented fears are common during adolescent development, as young people navigate the complexities of relationships, social expectations and identity outside of the family. For some adolescents, this can manifest as exaggerated perceptions of threat and fear in relation to others (e.g., Bird et al., 2019; Kingston et al., 2024). Paranoia (PA) describes exaggerated fears that others intend to cause one harm, with 20%–30% of UK adolescents reporting weekly paranoid ideation (e.g., Kingston et al., 2024). Social anxiety (SA) describes the fear of being humiliated, scrutinised or rejected by others. SA and PA are thematically similar, as well as sharing common predictor and maintenance factors (e.g., Newman‐Taylor & Stopa, 2013; Rietdijk et al., 2009; Schutters et al., 2012). However, some studies suggest they are nonetheless distinguishable constructs in adolescents (Kingston et al., 2024). The nature of their co‐development and their points of convergence and divergence during adolescence are largely unknown yet have the potential to greatly enhance our understanding of both, and of progression into serious mental illness (i.e., social anxiety disorder [SAD], schizophrenia).

Hierarchical models of PA, such as the Paranoia Hierarchy (Freeman et al., 2005), propose that common social anxieties (e.g., fears of rejection, feeling socially vulnerable) and ideas of reference (e.g., tendency to detect personal significance in otherwise neutral events) constitute a less pathological form of PA, and that persecutory beliefs build on these more commonly endorsed social fears. This implied developmental relationship of PA emerging from social evaluative concerns has been examined extensively in clinical and non‐clinical adult samples (e.g., Bebbington et al., 2013) but despite being a model of PA development, it has not been investigated in adolescence. Whilst there is no consensus on the definition of adolescence, it is flexibly defined as the onset of puberty to early adulthood. Here, we focus on mid‐to‐late adolescence (14–24 years). Investigating these associations in mid‐to‐late adolescence is important because: (1) adolescence is a unique phase of development, with multiple sensitive periods of brain development, and marked differences in social cognition relative to other life stages (e.g., Kilford et al., 2016; Kingston & Taylor, under review; Uhlhaas et al., 2023); (2) it is the life stage during which these fears typically become established and, for some, will escalate (Kessler et al., 2005); and (3) an increasing range of more clearly defined and differentiable mental health conditions emerge through adolescent development (Uhlhaas et al., 2023).

Existing research has shown that relative to age and sex‐matched adolescents without mental health problems, mid‐adolescents with SAD report elevated PA (Pisano et al., 2016). In a UK community mid‐adolescent sample, using a cross‐sectional design, Kingston et al. (2024) demonstrated that PA and SA were highly co‐morbid and that elevated levels of both was more common (21%) than either in isolation (SA only—14%, PA only—7%). Findings also indicated that SA and PA were related yet statistically distinguishable constructs, and that their co‐occurrence was associated with significantly greater emotional distress, relational anxiety, and a poor sense of school belonging relative to either one occurring in isolation. Using longitudinal methods in a late‐adolescent German sample (17–28 year), Schutters et al. (2012) showed that, at the symptomatic level, SA predicted subsequent onset of paranoid beliefs. However, when assessing the temporal association between paranoid symptoms and SA *disorder*, paranoid symptoms preceded SAD onset. Conflicting findings likely reflected the distinction between symptoms versus diagnosis, and limitations in the use of single endorsement of rare paranoid items as an index of paranoid symptomatology. PA was also only assessed when young people were 17+, thus limiting generalisability to younger cohorts. Interestingly, in line with Kingston et al. (2024) a distinguishing factor for PA versus SA in this sample was trauma exposure, alongside cannabis use.

In summary, there is good evidence to suggest that PA and SA co‐occur in adolescents, but there is insufficient longitudinal data about their co‐development. Investigating this has the potential to significantly advance our understanding of both types of experience in isolation, but also, critically, their developmental interplay. Symptoms may also hold predictive value for future diagnoses (Schutters et al., 2012). Based on the overarching hypothesis that PA emerges from socially anxious concerns, three pre‐registered hypotheses were tested using the Neuroscience in Psychiatry Network (NSPN) 2k cohort data.

1a. Structural differentiation: Replicating and advancing Kingston et al. (2024), two and three‐factor models distinguishing SA from ideas of reference and PA will demonstrate superior Confirmatory Factor Analysis (CFA) model fit to a single‐factor model. (b) Based on the increasing range of differentiable mental health symptoms and conditions through adolescence, we further predicted configural non‐invariance with increasingly better fit of the two and three factor models in older age groups.
2a. Temporal associations: In cross‐lagged panel analysis, SA at a given time‐point will be significantly associated with PA at the next time point. Additionally, (b) we also tested for the reverse temporal association competing with existing theoretical assumption, that is, whether PA significantly predicted SA at the subsequent time‐point.
3. Co‐developmental trajectories: Each author rated a set of possible co‐developmental trajectories as most to least likely based on the theoretical approaches of the hierarchical model of PA and previous studies to produce a predicted set of theoretically plausible trajectories to be compared with subsequent trajectory analyses (as they are exploratory in nature). Consensus amongst authors was reached through subsequent discussion. In descending order of highest to lowest likelihood, we hypothesised: (1) SA low and PA low (2) SA high and PA high (3) SA increasing and PA low (4) SA high and PA low (5) SA high and PA increasing (6) SA increasing and PA increasing (7) SA decreasing and PA low and (8) SA decreasing and PA decreasing.

## MATERIALS AND METHODS

### Design

We utilised the NSPN 2k cohort data. The NSPN was a multi‐centre accelerated longitudinal community cohort study, recruiting a normative adolescent to young adult sample of 14–24 years. Details of the full assessment battery are provided in Kiddle et al. (2018). In brief, a Home Questionnaire Pack and a Sociodemographic Questionnaire (SQ) together assessed participants' mood, behaviour and wellbeing along with demographic characteristics. Participants completed assessments at baseline, T2 (12‐months later, inter‐quartile range 11–14 months later, 70% response rate), and T3 (13 months later, inter‐quartile range 12–16 months later, 47% response rate). Sociodemographic characteristics of those who dropped out can be found in Kiddle et al. (2018), which reports that no systematic bias was observed. In the current study, based on pre‐registered hypotheses, we analysed Social Phobia and PA subscale data from the Schizotypal Personality Questionnaire (SPQ; Raine, 1991). All participants gave written informed consent to take part. For those under 16 years of age, consent was obtained from their legal guardian in addition to their own assent.

### Participants

Full participant recruitment methods and inclusion/exclusion criteria are detailed Kiddle et al. (2018). In brief, a sample of age and sex‐stratified 14–24 year olds were recruited from schools, colleges, National Health Service primary care services, and direct advertisement in north London and Cambridgeshire. Participants were evenly recruited from five age groups: 14–15, 16–17, 18–19, 20–21, and 22–24.99 years. 30,923 expression of interest forms were distributed within GP practices and schools, 4170 (13.5%) were returned to the research team, of which 3726 young people were eligible to participate and 2403 completed baseline assessments. At T2 and T3, a small subset of the sample (*n* = 288) received abridged versions of the questionnaire. Although various efforts were made (e.g., emailing study authors, contacting generic email address for NSPN, etc.), it was not possible to determine why this happened and therefore we decided to only include participants who received the full validated version of the SPQ. The final sample reported on in this manuscript, therefore, is *n* = 2115. Baseline demographic data, by age group, is reported in Table 1.

**TABLE 1:** Demographic (frequency) and SPQ (mean and SD) data split by age groups.

	Age group				
	14–15	16–17	18–19	20–21	22–24
*N*	401	447	416	408	443
Gender					
Female	55.11%	54.81%	55.05%	51.47%	56.21%
Male	44.89%	45.19%	44.95%	48.53%	43.79%
Ethnicity					
White	79.09%	69.53%	74.22%	79.66%	83.71%
Asian/Asian British	8.06%	13.32%	10.6%	8.33%	7.69%
Mixed	7.56%	7.67%	5.3%	5.39%	5.2%
Black/African/Caribbean/Black British	4.03%	7.22%	6.75%	2.94%	2.49%
Other	1.01%	2.03%	2.41%	2.45%	0.68%
Missing/decline to state	0.25%	0.23%	0.72%	1.23%	0.23%
Current occupation					
Full time education	61.56%	74.59%	74.22%	60.93%	37.78%
Working (part/full time)	1.04%	4.71%	16.39%	27.28%	50.9%
Unemployed	‐	1.65%	5.30%	6.64%	7.47%
Full time at home	‐	0.71%	0.24%	1.23%	0.45%
Other	0.26%	0.47%	1.20%	2.21%	2.26%
Missing/not applicable	37.14%	17.41%	2.65%	1.48%	1.13%
SPQ scores (*M*; SD)					
Social anxiety T1	3.94; 2.53	3.90; 2.47	4.04; 2.61	3.80; 2.54	3.64; 2.59
Social anxiety T2	3.51; 2.66	3.52; 2.56	3.72; 2.61	3.56; 2.57	3.53; 2.75
Social anxiety T3	3.37; 2.61	3.38; 2.69	3.83; 2.66	3.63; 2.71	3.56; 2.85
Ideas of reference T1	3.23; 2.53	3.08; 2.33	3.11; 2.35	2.62; 2.36	2.39; 2.31
Ideas of reference T2	2.35; 2.39	2.16; 2.29	2.39; 2.31	2.11; 2.27	1.88; 2.30
Ideas of reference T3	2.12; 2.29	1.81; 2.11	2.30; 2.33	1.75; 2.37	1.75; 2.34
Paranoia T1	2.72; 2.47	2.64; 2.23	2.83; 2.33	2.35; 2.24	2.37; 2.33
Paranoia T2	2.17; 2.42	2.04; 2.20	2.34; 2.28	2.02; 2.11	2.19; 2.29
Paranoia T3	1.80; 2.19	1.70; 2.01	2.33; 2.37	1.99; 2.26	2.09; 2.34

*Note*: Descriptive data on SPQ scores is based on completers, only.

Abbreviation: SPQ, Schizotypal Personality Questionnaire.

### Materials

Sociodemographic variables: Modelled on the 2011 public census data, the SQ was designed to assess family characteristics such as ethnicity, highest maternal and/or paternal qualification, current postcode, employment status etc. Comparing statistics to the 2011 census data, the NSPN Cohort: (1) broadly matched the ethnicity of the general population in England & Wales, with a slight over‐representation of mixed race and Asian participants; (2) closely resembled the England & Wales population structure in terms of UK versus non‐UK birth; (3) higher parental qualifications equating to 10% difference in achieving Level 1–4 qualification as compared to England & Wales consensus data yet vocational qualifications achieved did not notably differ. In terms of gender, (4) on average 5% more females and 5% fewer males completed the study relative to the consensus data and (5) compared to the distribution of Index of Multiple Deprivation ranks in England, the NSPN dataset had an under‐representation of those falling within the lowest 1st decile and an over‐representation within the ninth highest decile. The remaining deciles are broadly comparable to England.

The Schizotypal Personality Questionnaire (SPQ; Raine, 1991) is a 74‐item scale which measures nine subscales of schizotypy, of which the *ideas of reference* (9‐items), *excessive SA* (8‐items) and *suspiciousness* (8‐items) subscales were used in this manuscript. The SPQ was developed and validated to detect these symptoms in general population samples using dichotomised forced choice response options (‘Yes’/’No’). A higher sum score on subscales indicates more symptomology. The SPQ has previously evidenced high internal consistency (Cronbach's *α* = .91; Raine, 1991). In this sample, Cronbach's alpha for each subscale at baseline were .76 for SA, .75 for ideas of reference, and .81 for PA.

### Data‐analysis strategy

Since missing values were to be expected at follow‐up assessments due to attrition, potentially affecting the cross‐lagged panel and latent class growth analysis (LCGA), we imputed missing values for our main analyses. Based on the assumption of data missing at random (MAR), that is, missing values might be in part dependent on prior observed values of the data set (e.g., symptom levels), we used predictive mean matching to impute missing SPQ‐subscale scores based on the closest match in existing values (Asendorpf et al., 2014). All imputations were made at the subscale score level. Additionally, we used the reduced sample without imputed missing values for a sensitivity analysis of our cross‐lagged panel and LCGA results, in order to transparently document if there were any deviations in result from the imputation procedure. For all significance tests, we used the significance level of *p* < .05. This is because the number of tests were pre‐established and registered prior to data‐analyses, thus ensuring a limit on the number of tests and capping potential biases due to alpha‐error inflation. In addition, our analyses involved comparing competing temporal pathways, including hypothesised and reverse‐direction effects. Applying more conservative corrections would have reduced statistical power for individual path coefficients and may have disproportionately increased the risk of Type II errors, particularly for reverse‐lagged effects that were not expected to support our hypotheses (e.g., when testing for reverse time‐lagged effects of PA on subsequent SA).

#### Hypothesis 1

For our factor analyses, we tested one, two (SA vs. PA plus ideas of reference) and three factor (SA, reference, PA) models. We expected skewed, non‐normal distributed data, thus we preregistered our confirmatory factor analyses to be conducted with maximum likelihood estimation with robust (Huber‐White) standard errors and a scaled test statistic asymptotically equal to the Yuan‐Bentler test statistic (to allow for the inclusion of incomplete data). However, since the answering format for the SPQ was dichotomous, we also added an estimation using unweighted least squares. The model fit was evaluated using the indices Comparative Fit Index (CFI), Root Mean Square Error of Approximation (RMSEA), and Standardised Root Mean Square Residual (SRMR). First, we calculated the full sample and configural invariance models; established thresholds for the aforementioned indices were utilised (i.e., CFI: good fit CFI > 0.95, sufficient fit CFI > 0.90; RMSEA: good fit RMSEA < 0.05, sufficient fit RMSEA < 0.08; SRMR good fit at SRMR < 0.08; Hu & Bentler, 1999). Next, exploratory evaluation of metric and scalar invariance was conducted and evaluated by differences in fit‐indices between two consecutive invariance steps, as established by Meade et al. (2008): Metric invariance cutoffs were ΔCFI > −0.010, ΔRMSEA < 0.015, and ΔSRMR < 0.030; scalar invariance cutoffs were ΔCFI > −0.010, ΔRMSEA < 0.015, and ΔSRMR < 0.015.

#### Hypothesis 2

For the cross‐lagged panel analysis, sum‐scores of the SPQ‐subscales SA and PA were calculated for each participant at each of the time‐points. For the cross‐lagged panel, we set the unstandardised regression coefficients for corresponding predictions to be equal between T1–T2 and T2–T3. Standardised beta‐scores were calculated for evaluating the difference between cross‐lagged associations. In principle, random intercept cross‐lagged panel models would be a preferable option for these analyses (Lucas, 2023), since they can distinguish stable trait components and their co‐variation from the time‐lagged influence on state changes in the analysed variables. However, in this case, we were faced with a fixed low number of three assessments per person and a per‐determined sample size which we assumed to likely result in insufficient test‐power (see Mulder, 2023), so we opted to use standard cross‐lagged panel models for more stable and reliable estimation.

#### Hypothesis 3

For the LCGA, we expected a range of 4–10 different groups based on prior cross‐sectional findings reported in Kingston et al. (2024). Furthermore, based on our prior work, all authors independently ranked what they perceived to be the most likely co‐developmental trajectories and through subsequent discussion, consensus was reached and reported in the pre‐registration (OSF Registries | The structure of paranoia, ideas of reference and social anxiety in adolescents). To determine the number of classes, we calculated a series of latent class growth curve models (LCGA) of PA and SA, with full information maximum likelihood estimation. The lower end of potential classes was 3, and the upper end was 10 classes. The models were evaluated by comparing the indices AIC, adjusted BIC, and entropy, and by testing for the information value of an additional category with the Vuong‐Lo‐Mendell‐Rubin Test. Classes were then described by their intercepts (i.e., starting values of SA, and PA), as well as whether the slope parameters for SA or PA were significantly above 0 (increasing SA/PA), below 0 (decreasing SA/PA) or non‐significant (stable SA/PA).

## RESULTS

Descriptive statistics for SA, ideas of reference and PA, by age and across time points are reported in Table 1. Means suggest overall higher SA than PA scores. Age differences are most apparent for ideas of reference, with higher scores in the younger cohort and reducing with age and measurement point. These trends were not reflected (SA) or notably less marked (PA) in the other two subscales. Published norms from the validation university sample (Raine et al., 1991) suggested comparable SA scores, but lower PA and ideas of reference in the current as compared to validation sample. However, mean SA and PA scores are comparable to a larger undergraduate sample (Fonseca‐Pedrero et al., 2014), with ideas of reference lower again.

### Hypothesis 1a and 1b: Factor analysis

The results of all CFA are summarised in Figure 1. As depicted, the models distinguishing SA and PA (or PA and ideas of reference) showed sufficient fit by two of the three indices in both variations of the estimation. Contrary to hypothesis 1b, the models distinguishing SA from ideas of reference/PA (with the three‐factor model showing slightly better fit than the two‐factor model) showed better model fit across all age groups, with invariance analysis fit indices roughly at the same level as individual age group fit indices. Thus, there was no evidence for increasing differentiation between SA and PA in older cohorts. Finally, within the confines of the mixed model‐fit results, all differences in fit indices were within the thresholds for scalar and metric invariance (metric invariance: all ΔCFI > −0.010, ΔRMSEA < 0.015, and ΔSRMR < 0.030; scalar invariance: all ΔCFI > −0.010, ΔRMSEA < 0.015, and ΔSRMR < 0.015).

**FIGURE 1:**
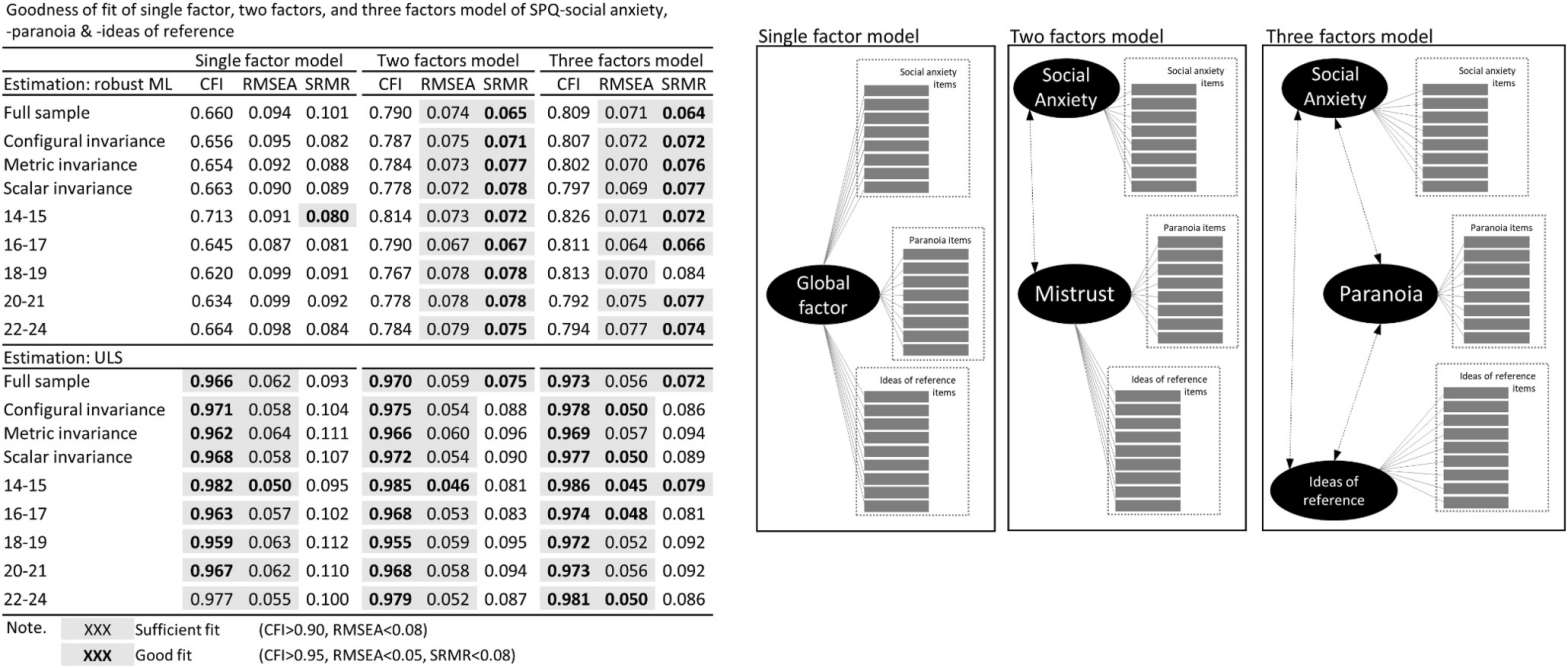
Model depiction and results summary of the confirmatory factor analyses.

### Hypothesis 2a and 2b: Cross‐lagged longitudinal model

Results of the cross‐lagged longitudinal models are summarised in Table 2. As can be seen, the single group structural equation models showed larger standardised regression coefficients for SA at Tx predicting PA at Tx + 1 than vice versa. This was true for the main analysis and the sensitivity analysis without the imputed missing values. Further, exploratory analysis differentiating the sample by the five NSPN age groups showed that the most consistent differences were between the oldest and youngest subgroups: In the 22‐ to 24‐year‐olds, standardised coefficients for SA predicting subsequent PA were consistently significant across all analyses, whereas the corresponding coefficients for PA predicting subsequent SA were lower, by comparison, and non‐significant. For the 14‐ to 15‐year‐olds this pattern was reversed, so that PA significantly predicted subsequent SA, whereas the connections from SA to PA were smaller and consistently non‐significant. For most intermediary groups (16–17, 18–19, 20–21), the hypothesised pattern of SA to PA exceeding PA to SA paths was found by descriptive value, with the exception being the 20‐ to 21‐year‐olds: here the reversed pattern was found in the main analysis (but not the sensitivity analysis without imputed missing values).

**TABLE 2:** Results (main and stability analysis) from the cross‐lagged panel analysis of SA and PA.

Group	T1–T2					T2–T3					Covariance SA‐PA			Fit indices			
	SA → PA	PA → SA	Δ*β*	SA → SA	PA → PA	SA → PA	PA → SA	Δ*β*	SA → SA	PA → PA	T1	T2	T3	CFI	TLI	RMSEA	SRMR
Main analysis (full sample with missing values imputed using predictive mean matching)																	
All	**0.083**	**0.073**	0.010	0.662	0.630	**0.091**	**0.075**	0.016	0.698	0.677	0.422	0.381	0.373	0.965	0.935	0.120	0.043
Subgroups in multigroup SEM														0.961	0.927	0.129	0.043
14–15	0.045	**0.105**	−0.060	0.596	0.640	0.051	**0.105**	−0.054	0.635	0.683	0.423	0.440	0.420				
16–17	**0.065**	**0.054**	0.011	0.640	0.588	**0.077**	**0.060**	0.017	0.702	0.698	0.368	0.298	0.354				
18–19	**0.110**	**0.070**	0.040	0.657	0.713	**0.076**	**0.123**	−0.047	0.600	0.664	0.401	0.407	0.326				
20–21	**0.085**	**0.106**	−0.021	0.636	0.657	**0.090**	**0.108**	−0.018	0.660	0.682	0.433	0.399	0.363				
22–24	**0.102**	0.048	0.054	0.768	0.670	**0.106**	0.046	0.060	0.763	0.672	0.475	0.372	0.386				
Stability analysis (including only participant answers as reported with no missing value imputation)																	
All	**0.079**	**0.065**	0.014	0.669	0.625	**0.086**	**0.065**	0.021	0.691	0.655	0.427	0.389	0.356	0.964	0.932	0.121	0.04
Subgroups in multigroup SEM														0.959	0.924	0.129	0.047
14–15	0.042	**0.084**	−0.042	0.589	0.623	0.048	**0.084**	−0.036	0.629	0.657	0.428	0.446	0.415				
16–17	0.048	0.011	0.037	0.656	0.581	0.056	0.011	0.045	0.695	0.631	0.379	0.264	0.396				
18–19	**0.108**	**0.097**	0.011	0.649	0.608	**0.119**	**0.106**	0.013	0.690	0.686	0.403	0.411	0.305				
20–21	**0.096**	**0.091**	0.005	0.656	0.629	**0.101**	**0.087**	0.014	0.653	0.632	0.434	0.398	0.261				
22–24	**0.088**	0.053	0.035	0.782	0.683	**0.091**	0.050	0.041	0.771	0.676	0.479	0.440	0.352				

*Note*: All path coefficients are standardised scores. A positive Δ*β* signifies a higher association between SA and subsequent PA than vice versa. Significant cross‐lagged pathways (*p* < .05) are printed in bold.

Abbreviations: CFI, Comparative Fit Index; PA, paranoia; RMSEA, Root Mean Square Error of Approximation; SA, social anxiety; SRMR, Standardised Root Mean Square Residual; TLI, Tucker–Lewis Index.

### Hypothesis 3: Latent growth cluster analysis

Latent growth cluster analysis in the full sample with imputed missing values showed optimal fit based on the VLMR‐Test at six categories (see Table 3), with no additional significant benefit of a seventh or eighth class. Entropy remained roughly the same, regardless of the number of classes tested, whereas Akaike Information Criterion (AIC) and adjusted Bayesian Information Criterion (BIC) decreased continuously with an increased number of classes, with this trend exceeding the upper end of the test range of up to 10 classes. Thus 6 classes were determined as the optimal number in the main analysis.

**TABLE 3:** Identification of the number of classes for latent growth based on AIC, adjusted BIC, entropy, and VLMR likelihood ratio test.

Number of classes	Main analysis (full sample with missing values)						Stability analysis (data as assessed with no imputation)					
	Entropy	AIC	Adj. BIC	*p* (VLMR‐test)	Range av. classification likelihood	Range class size, %	Entropy	AIC	Adj. BIC	*p* (VLMR‐test)	Range av. classification likelihood	Range class size, %
3	0.88	52,910	52,960	<.001	0.91–0.96	21.1–52.3	0.78	39,526	39,575	<.001	0.84–0.93	19.2–54.3
4	**0.89**	52,047	52,109	<.001	0.89–0.96	9.2–47.7	**0.79**	39,063	39,125	.027	0.78–0.91	10.0–47.7
5	0.84	51,686	51,760	<.001	0.81–0.95	8.9–35.8	0.74	38,816	38,890	**.014**	0.74–0.89	10.5–35.5
6	0.84	51,375	51,462	**.025**	**0.81**–**0.93**	**7.9**–**35.0**	0.75	38,656	38,743	.271	0.74–0.89	3.5–34.9
7	0.85	51,170	51,270	.154	0.88–0.93	3.9–33.8	0.75	38,476	38,576	.027	0.68–0.89	5.6–32.1
8	0.84	51,001	51,113	.104	0.78–0.93	3.7–33.4	0.76	38,330	38,442	.332	0.68–0.87	4.2–31.8
9	0.85	50,819	50,943	.042	0.79–0.92	3.5–32.0	0.75	38,208	38,332	.476	0.68–0.87	3.9–31.3
10	0.84	**50,719**	**50,856**	.407	0.78–0.93	3.0–30.1	0.75	**38,110**	**38,246**	.681	0.65–0.87	2.8–31.4

*Note*: Positive indicators for the respective class‐number are printed in bold.

Abbreviations: AIC, Akaike Information Criterion; BIC, Bayesian Information Criterion; VLMR, Vuong‐Lo Mendell‐Rubin.

An overview of the six classes is shown in Figure 2. As expected, the largest class comprised more than a third of the sample and was defined by low levels of SA & PA (low + descending, see Figure 2). Partially consistent with expectation, the next two largest classes were defined by increased SA and low values of PA. The fourth class is characterised by high levels of SA that further increase over time and stable levels of elevated PA. Surprisingly, we found one group with SA descending over time, but PA levels remaining elevated. The smallest group showed stably high levels of both SA and PA, which we had anticipated to be more common.

**FIGURE 2:**
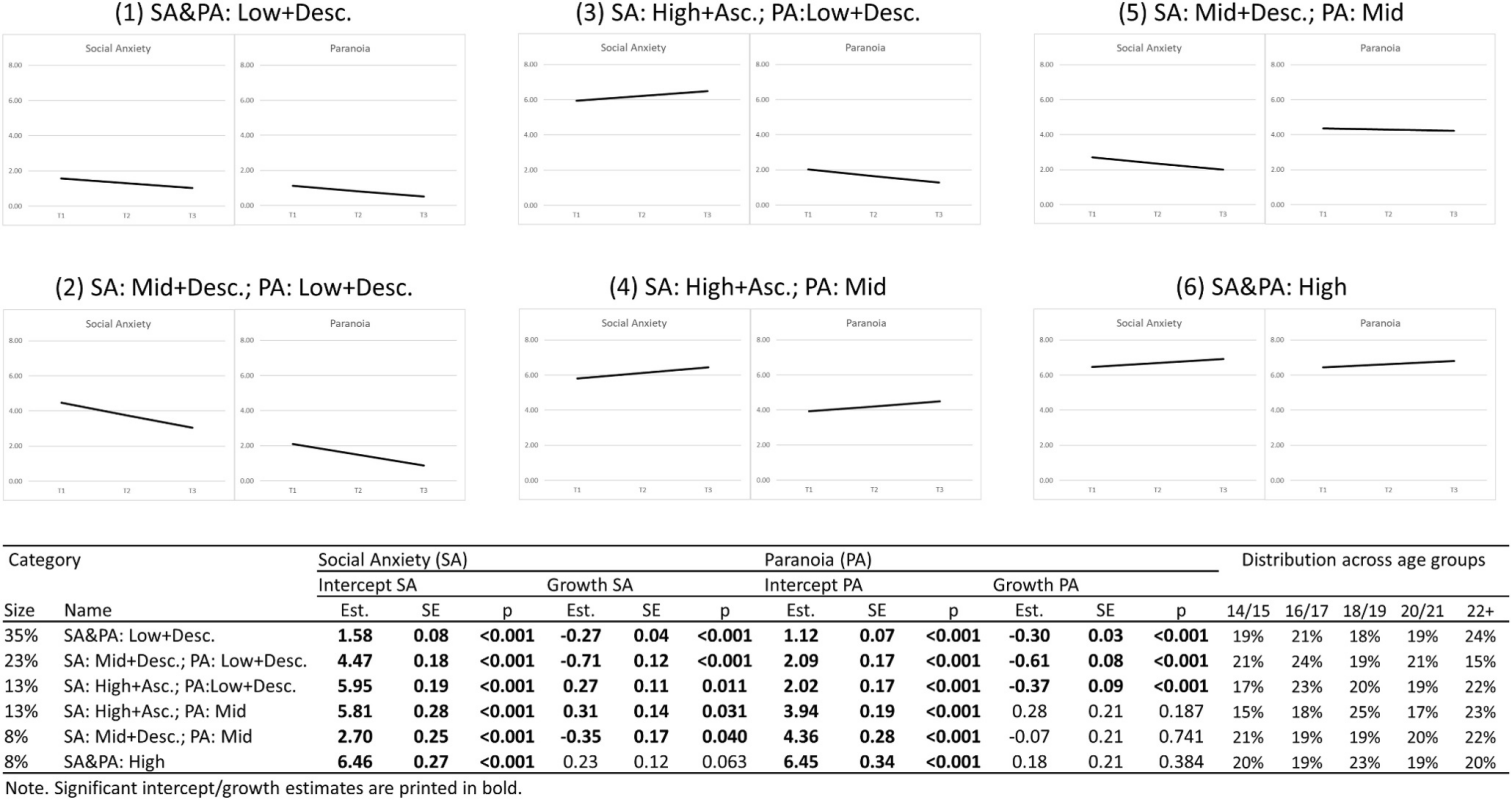
Overview of six classes latent growth curve cluster analysis (main analysis), predicted course of paranoia and social anxiety (Schizotypal Personality Questionnaire‐sum scores) over time by class.

Comparing these results with the clusters identified in our sensitivity analyses showed the three largest groups in both analyses were identical in their respective patterns (SA & PA low, SA at mid level & PA low, SA high and PA low, see Supporting Information S1: Figure S1), with most participants allocated to corresponding classes in both the main and stability analysis (see Supporting Information S1: Table S1). The fourth largest class in the stability analysis was characterised by high SA and high PA and comprised most of the high SA & high PA class from the main analyses as well as the largest group of the high/ascending SA & stable/elevated PA group. Finally, we again found a small group characterised by decreasing SA and stable PA. Comparing the distribution of the six classes across the age groups yielded no indication of substantial differences across age groups (see Figure 2).

## DISCUSSION

This study had three main aims. Following on from Kingston et al. (2024), we sought to: (1) replicate the differentiation of SA and PA via measurement models, with non‐invariance over age groups and increasing differentiation in older adolescents; (2) test the hypothesis that SA will precede and predict PA, but not vice versa; and (3) assess co‐developmental trajectories over time. A priori predictions were made, based on cross‐sectional data and aforementioned theorising (Kingston et al., 2024), anticipating that the most common trajectory would be low scores on both, followed by high on both. Next, we expected (in order) SA increasing and PA low, SA high and PA low, SA high and PA increasing (i.e., for all three, SA as high/increasing prior to PA). Next, we anticipated both increasing, SA decreasing and PA low, and finally both decreasing. Consistent with the PA hierarchy, we did not anticipate PA in the absence of SA to emerge as a common cluster.

Firstly, consistent with Kingston et al. (2024), we replicated the differentiation of SA and PA; the two and three factor model, with SA and PA as related but separate constructs, demonstrated superior fit statistics relative to a single factor model, across all age groups (with the three‐factor model showing slightly superior fit to the two‐factor model). This replication is notable, given that the two studies employed different self‐report measures *and* that the current study used items for SA and PA that came from the same questionnaire. In Kingston et al., we could not rule out the possibility that the differentiation between SA and PA, observed in the measurement models, was an artefact of using two different questionnaires. This study also used a more sociodemographically representative sample that spanned mid‐to‐late adolescence (13–22 vs. 14–17 years), thus enhancing confidence in the true distinction between the two constructs, as well as in the generalisability of the findings. Contrary to prediction, we did not show any age effect; that is, there was no evidence for increasing differentiation between the constructs in older cohorts. Based on the current findings, therefore, SA and PA seem to be equally distinguishable across mid‐to‐late adolescence. These findings are consistent with research on adult populations, showing that PA and SA share common correlates but nonetheless have points of differentiation and distinction (Freeman et al., 2008; Freeman & Loe, 2023; Stopa et al., 2013).

Secondly, cross‐lagged panel analysis showed that SA was a more significant predictor of subsequent (approximately 1 year later) PA than vice versa. This finding is consistent with and extends previous research showing that anxiety manipulations increase PA (Bennetts et al., 2022) and that SA predicts subsequent PA in late adolescence/early adulthood (Schutters et al., 2012). Interestingly, a more nuanced account emerged when examining patterns across age groups. Here, different patterns emerged at the lower and upper ends of the age range. In the 22–24 year olds, there were consistently higher associations from SA to PA, whereas in the youngest group (14–15 year olds), this pattern was reversed: PA significantly predicted subsequent SA, whereas the connections from SA to PA were smaller and non‐significant. It is important not to over‐interpret age differences, given the small samples and lack of a priori age‐specific hypotheses. However, findings suggest that endorsing paranoid beliefs at a younger age (i.e., 14/15) may set the scene for increased susceptibility for SA at 16/17 years old, whereas SA in 16+ age groups increased susceptibility for future PA, with some evidence of reciprocal associations (i.e., significant associations in both directions). The *PA‐fuelling‐SA* pathway could reflect a younger group of adolescents whose paranoid beliefs are routed in early experiences, such as parent/carer relationships (e.g., insecure attachments to parents/carers), developmental trauma, cultural mistrust and/or lived experiences of harm from others, which set the scene for subsequent social anxieties through mid‐late adolescence (see Bloomfield et al., 2021). Conversely, consistent with our a priori theorising, the second pathway of *SA‐fuelling‐PA* suggests a more common pattern whereby feeling socially anxious increases susceptibility to future PA. Interestingly, Kingston et al. (2024) reported that participants (14–17 years) with high PA (with or without SA) were significantly younger than those with high SA only. Future research should explore age differences further, including an examination of sociodemographic, cognitive, and social characteristics that define this early *PA‐fuelling‐SA* pathway. Additionally, whilst we used age as a proxy for development, future research could utilise measures that are more indicative of developmental stage, such as pubertal stage, cognitive ability or a composite of biological and social cognitive variables. Finally, qualitative accounts of how PA and SA converge and diverge across adolescent development are likely to enrich our understanding of the nuances of their association (e.g., see Stopa et al., 2013).

Thirdly, as expected, the most common co‐developmental profile was low levels of SA and PA, which was evenly distributed across ages, but most common to the eldest group. This group accounted for 35% of the full sample, which is notably smaller than the corresponding cross‐sectional group in Kingston et al., where the low‐low group accounted for 59% of the sample. However, given that the second largest class (22%) also started at low‐low with SA then increasing over time, the overall number of low‐low participants at baseline (35% + 22% = 57%) corresponded closely to the cross‐sectional ‘snapshot’ in Kingston et al. (2024). This similarity is particularly interesting given the differences in the measures and analysis between these two studies; in Kingston et al., a high threshold for caseness was set, whereby ‘at least severe’ levels of PA were required to be classified as elevated levels. Conversely, here, no thresholds were set and instead clusters were mapped descriptively to low/mid/high levels. We expected the next most common cluster to be high‐high, but instead it was middle and descending levels of SA with low and descending levels of PA (23%), more common to 16–17 and 20–21 year olds and notably less common in the eldest group. Thereafter, two clusters of equal prevalence (13%) were identified, both characterised by high and ascending SA alongside either low and descending or mid and constant levels of PA. The both‐high group was less common than anticipated, occurring in only 8% of cases and appearing to be equally common across age groups although with a slight increase in prevalence at 18–19 years old. This contrasts to 21% in the Kingston et al. (2024) paper, but could just reflect differences in categorisation (i.e., elevated or not elevated vs. graded levels of low/medium/high in the current paper).

Across findings, there were minimal differences between main analyses with imputation versus the stability analyses using data as recorded only and these were within the assumptions of MAR attrition. Specifically, the largest notable difference of a loss of one elevated/increasing category in stability versus main analyses fits with the idea that some cases of attrition are possibly attributed to higher symptom levels.

Two replicated findings are also of note. Both here and in Kingston et al. (2024), a small (7%) group of adolescents reported elevated PA without/with low SA. Kingston et al. showed that this group had the highest exposure to bullying, discrimination and adverse life events but also reported a greater sense of belonging, less loneliness and inferiority concerns, and less avoidance in relationships relative to the high SA and both‐high group. This could represent a group of adolescents who hold fears about particular others, perhaps arising from specific life experiences, but who nonetheless function well socially and sustain relationships. This replicated effect warrants further examination and raises an interesting possibility to study paranoid beliefs that may not impact social relationships and that may not involve avoidance behaviours commonly associated with SA. Further examination of the psychosocial characteristics of this subgroup is an important next step. The other replication is the co‐occurrence of SA and PA. In this study, in 21% of cases, stably elevated SA was accompanied by elevated (mid/high) PA, which can be compared to 21% in Kingston et al. (2024). Together, these findings underscore the importance of measuring, understanding, and addressing where implicated, PA in adolescents presenting with SA.

Findings should be considered in the light of some limitations. The use of only three assessment points, over 2 years, lacks sensitivity to shifts in symptoms that occur with greater frequency. Compared to adults, adolescents undergo rapid and dynamic changes in social, emotional, behavioural, and cognitive development and functioning (e.g., Dahl et al., 2018). More frequent assessments would track changes with greater precision and sensitivity to change. Further, they would also allow for more sophisticated model estimation given the similar overall sample size, for example, random intercept cross lagged panel models, that would allow to distinguish and separately estimate stable trait components and dynamic changes in symptom dimensions. The sole use of self‐report is also a key limitation, especially for the case of paranoid beliefs. Self‐report measures benefit from easy dissemination, yet failed to capture unfoundedness, which is a critical and defining feature of PA. Future research would benefit from combining self‐report assessment with more objective assessment of PA, for example, through the use of virtual reality methods. Equally, the accuracy with which mental health can be assessed within adolescents is greatly improved by multi‐informant data, and findings from this study relate only to the adolescents' self‐reports. Finally, the SPQ assesses SA and PA dichotomously (yes/no) rather than continuously. This enforces a categorical structure to these experiences, which is counter to prevailing theories of a continuum of experience and qualitative reports of the experience of PA (Boyd & Gumley, 2007).

Despite limitations, the findings have important implications. Future research tracking the co‐development of SA and PA through adolescence with greater precision, and by simultaneously assessing risk factors and causal mechanisms, is a critical next step. This should include more frequent assessments, using continuous (rather than dichotomous) measurement and, critically, with the capacity to determine the *unfoundedness* of paranoid beliefs. Determining the *impact* of different trajectories on key adolescent outcomes (e.g., well‐being, school attendance and attainment, transition into adulthood, presence or absence of a mental health condition) is also imperative. Indeed, ascertaining what level of SA and PA is problematic for young people is a key next step. Although more established for SA, for the case of PA, the point at which fears are best categorised as ‘PA’ versus a natural spike in interpersonal worries is still unclear. Future psychometric research should determine thresholds using samples with established clinical diagnoses.

## CONCLUSIONS

In summary, our findings show that SA and PA are commonly comorbid in adolescence. Furthermore, the overall pattern of findings was most consistent with the *SA‐fuelling‐PA* hypothesis. This emerged as the prevailing pathway in the cross‐lagged and cluster analysis, where elevated SA was more common than elevated PA, and SA was more often elevated *prior to* PA. However, findings also detected a *PA‐to‐SA* pathway in the youngest age group and whilst *SA‐fuelling‐PA* was the most significant pathway, reciprocal associations were also observed.

## AUTHOR CONTRIBUTIONS

**Jessica L. Kingston**: Conceptualization; investigation; writing—original draft; methodology; project administration. **Björn Schlier**: Conceptualization; investigation; methodology; formal analysis; visualization; writing—review and editing. **Richard P. Bentall**: Conceptualization; investigation; writing—review and editing; methodology. **Christopher D. J. Taylor**: Conceptualization; investigation; methodology; writing—review and editing. **Eleanor Leigh**: Conceptualization; investigation; methodology; writing—review and editing; supervision.

## CONFLICT OF INTEREST STATEMENT

The authors declare no conflicts of interest.

## ETHICAL CONSIDERATIONS

All participants gave written informed consent to take part. For those under 16 years of age, consent was obtained from their legal guardian in addition to their own assent. The NSPN 2k project received approval from the Cambridge Central Research Ethics Committee. Ethical review and approval for this project were not sought because all the research data used in the article were sourced from external, publicly available databases.

## AI STATEMENT

The Authors declare that they have not used AI in any capacity that would require disclosure in accordance with Wiley's AI Guidelines, nor in any capacity that would reasonably require discloser for editors, reviewers and readers to properly evaluate their research or manuscript. The authors confirm that AI has not been used for purposes including but NOT limited to drafting and editing, to generate substantial text or restructure arguments, nor in the research methodology. The authors take full responsibility for the accuracy of this statement.

## Supporting information

Supporting Information S1

## Data Availability

The NSPN 2k data that support the findings of this study is available in NSPN at https://nspn.org.uk/, upon request.
