# Supplementary material for: Paranoia and social anxiety: Structural and temporal relationships across adolescence
Source: JCPP Adv. 2026 Oct 12:e70181. Online ahead of print. doi: 10.1002/jcv2.70181 (PMC13658955; doi:10.1002/jcv2.70181)
Supplement: Supplementary file 1 [file JCV2-9999-e70181-s001.docx]

**Paranoia and Social Anxiety:**

**Structural and Temporal Relationships Across Adolescence**

**Supporting Information**

Figure S1

Overview of five classes latent growth curve cluster analysis (stability analysis with data as assessed) predicted course of paranoia and social anxiety (SPQ-sum scores) over time by class

Table S1

Overlap in class allocation between six classes main analysis and five classes stability analysis.

| Category |  | SA&PA: Low+Desc. | SA: Mid+Desc.; PA: Low+Desc. | SA: High; PA: Low+Desc. | SA&PA: High | SA: Mid/Low+Desc.; PA: Mid |
| --- | --- | --- | --- | --- | --- | --- |
| SA&PA: Low+Desc. | N | **661** | 58 | 1 | 0 | 16 |
|  | % of row sum | **89.8%** | 7.9% | 0.1% | 0.0% | 2.2% |
|  | Imputed values | **25.3%** | 30.7% | 66.7% |  | 56.3% |
| SA: Mid+Desc.;  PA: Low+Desc. | N | 51 | **342** | 59 | 9 | 30 |
|  | % of row sum | 10.4% | **69.7%** | 12.0% | 1.8% | 6.1% |
|  | Imputed values | 44.44% | 17.0% | 42.1% | 59.3% | 30.0% |
| SA: High+Asc.;  PA:Low+Desc. | N | 6 | 45 | **216** | 6 | 2 |
|  | % of row sum | 2.2% | 16.4% | **78.5%** | 2.2% | 0.7% |
|  | Imputed values | 63.9% | 40.4% | 14.7% | 61.1% | 66.7% |
| SA: High+Asc.;  PA: Mid | N | 10 | 19 | 75 | **125** | 36 |
|  | % of row sum | 3.8% | 7.2% | 28.3% | **47.2%** | 13.6% |
|  | Imputed values | 70.0% | 58.8% | 23.8% | **23.2%** | 19.4% |
| SA: Mid+Desc.;  PA: Mid | N | 19 | 11 | 5 | 15 | **124** |
|  | % of rowsum | 10.9% | 6.3% | 2.9% | 8.6% | **71.3%** |
|  | Imputed values | 43.0% | 50.0% | 60.0% | 60.0% | **20.0%** |
| SA&PA: High | N | 1 | 1 | 6 | **146** | 14 |
|  | % of row sum | 0.6% | 0.6% | 3.6% | **86.9%** | 8.3% |
|  | Imputed values | 66.7% | 66.7% | 66.7% | **26.4%** | 54.8% |

Note. Cells with largest
